# Immediate occlusal vs nonocclusal loading of implants: A randomized prospective clinical pilot study and patient centered outcome after 36 months

**DOI:** 10.1111/cid.12770

**Published:** 2019-05-07

**Authors:** Susanne Vogl, Marlene Stopper, Markus Hof, Kerstin Theisen, Walther A. Wegscheider, Martin Lorenzoni

**Affiliations:** ^1^ Division of Operative dentistry, Periodontology and Prosthodontics, Department of Dental Medicine and Oral Health Medical University of Graz Graz Austria; ^2^ Department of Oral Surgery, Dental Clinics Faculty of Medicine at the Sigmund Freud University Vienna Austria

**Keywords:** bone loss, computer‐assisted, immediate function, immediate loading, marginal bone loss, patient satisfaction, randomized controlled trial

## Abstract

**Background:**

Immediate provisionalization reduces chair time and improves patient comfort.

**Purpose:**

To analyze immediate functional loading vs nonfunctional loading with restorations in the posterior mandible for marginal bone defects, implant success/survival, and patient satisfaction.

**Materials and Methods:**

A randomized controlled clinical trial was designed to assess these parameters based on 20 adult patients who underwent implant surgery, followed by immediate delivery of screw‐retained or cemented single or splinted restorations in full occlusal contact or in infraocclusion (test and control group). A questionnaire with visual analog scales was used to assess patient satisfaction.

**Results:**

Following 36‐month data were evaluable for 9 patients (21 implants) in the study group (immediate functional loading) and for 10 patients (31 implants) in the control group (immediate nonfunctional loading). One implant in the control group was lost, hence the overall implant survival and success rate was 98.2%. Marginal bone defects were consistent with previous studies and comparable in both groups. Periotest values did not significantly change from baseline and the 12‐month follow‐up (Friedmann test). Patient satisfaction was high and did not involve any significant intergroup differences (Mann‐Whitney *U*‐test).

**Conclusions:**

Both types of immediate provisional restorations are viable in selected patients. Larger randomized controlled trials are needed to establish immediate functional loading as a standard treatment for partially edentulous jaws.

## 1 INTRODUCTION

Current strategies of implant dentistry are aimed at minimizing surgical interventions and postoperative discomfort while improving patient satisfaction regarding function and esthetics.^1^ Immediate loading was introduced against this background, has since been extensively discussed in the literature, and has been found to be a valid strategy of treatment offering implant survival rates of 95% to 98.8% in the posterior mandible^2^, ^3^, ^4^ if appropriate patient selection is ensured.^5^, ^6^, ^7^, ^8^, ^9^, ^10^, ^11^ In addition to these clinical parameters, patient satisfaction and well‐being is another major criterion of successful implant treatment. We have devoted attention specifically to this topic,^12^ but generally speaking, little data continues to be available on patient satisfaction regarding esthetics, masticatory function, access for oral hygiene, as well as restorative maintenance requirements.

Immediate loading in partially edentulous mandibles is today considered viable in the hands of experienced clinicians.^13^, ^14^ Forty implants, inserted and immediately loaded to replace lower molars, were found to yield a 5‐year survival rate of 95% and a mean crestal bone loss of 1.17 mm.^15^ Immediate occlusal loading of 139 implants yielded a cumulative survival of 99% after 1 year, with a mean of 1.01 mm in marginal bone resorption.^11^ Immediate loading of 143 implants led to a 94% cumulative survival after 1 year in function, involving a mean crestal bone loss of 0.33 mm after conventional vs 0.24 mm after flapless implant surgery, which did not seem to make a difference.^9^ Immediate loading yielded a 12‐month success rate of 97.5% based on 40 implants supporting splinted restorations in 20 patients with missing mandibular premolars and molars.^16^ In addition, a recent review has disclosed no significant differences in implant survival, marginal bone loss, and mechanical or biological complications between immediately and conventionally loaded single implants in the posterior mandible.^17^

Nonfunctional protocols of immediate loading have been introduced so as to protect newly inserted implants from exposure to any excessive functional or parafunctional forces in partially edentulous patients,^18^ as complications like bruxism and severe clenching have been suspected to increase the risk of failure among immediately loaded implants.^19^ Studies have reported lower implant survival rates after immediate functional loading than after both immediate nonfunctional restoration and delayed loading.^20^, ^21^ Other authors did not observe any differences between immediate functional and nonfunctional loading with regard to implant survival, bone loss, or soft‐tissue healing.^4^, ^10^, ^11^, ^22^

Hence the aim of the present randomized controlled clinical trial was to assess marginal bone defects (MBDs), implant success and survival as well as patient satisfaction associated with immediate functional vs immediate nonfunctional loading of posterior implants in partially edentulous patients. This report covers an observation period of 36 months.

## 2 MATERIALS AND METHODS

### 2.1 Design and pilot study

The design of this randomized controlled clinical trial was approved by the institutional review board (ethics commission) at Medical University of Graz (ref: 23‐202 ex 10/11 and 27‐237 ex 14/15). The study was conducted in accordance with both the ICH‐GCP Guidelines for Clinical Trials and the Declaration of Helsinki as revised in 2008. All patients included gave their informed consent after being comprehensively informed about the study, the clinical parameters included in the analysis were described in a previous report of the 12‐month data, which was published as a pilot study.^23^

### 2.2 Patient enrollment

Twenty patients, all treated at our center exclusively, were enrolled between 3/2011 and 4/2012. A total of 59 implants were initially planned. Each patient was screened by reviewing his or her medical history, obtaining a panoramic radiograph, as well as an alginate impression (Xantalgin select; Heraeus Kulzer, Hanau, Germany). Only adult patients showing partial edentulism in posterior segments with no need for extensive grafting (at least 5 mm in width and 10 mm in height) were included in the study. Minimum primary intraoperative stability for impressioning was determined with more than 20 Ncm. Heavy smokers (>10 cigarettes a day), patients with active local inflammation or metabolic disease were excluded. Also patients with a history of irradiation or chemotherapy in the head‐and‐neck area, use of bisphosphonates, pregnancy, or evidence of severe parafunction (bruxing/clenching) were excluded.

### 2.3 Group assignment

An independent examiner randomized each patient to the test or the control group prior to laboratory fabrication, using the tool “Randomizer for Clinical Trials” provided by our Institute for Medical Informatics, Statistics and Documentation (randomizer.at). Immediate restorations were to be used, adjusted either to full occlusal loading by shimstock (test group) or to infraocclusion in maximum intercuspidation (control group).

### 2.4 Planning and medication

An experienced clinician planned all restorations, using a prosthetic‐driven approach. For details on the 3D implant planning, the reader is referred to our pilot study.^23^ Three dimensional planning was based on computed tomography and cone beam computed tomography. Each restorative treatment plan was verified for whether it was consistent with the patient's anatomy and location of sensitive structures by using 3D implant planning software (Simplant Crystal; Materialize Dental, Leuven, Belgium). Patients were instructed to rinse with chlorhexidine digluconate 0.2% for 1 minute prior to surgery, the latter being performed under local anesthesia (Ultracain dental forte; Sanofis‐Aventis, Vienna, Austria). Antibiotic treatment was started 1 day before surgery and carried on for 5 days (Augmentin 1 g twice daily; Smithkline Beecham, Worthing, UK).

### 2.5 Implant placement

**Figure 1:**
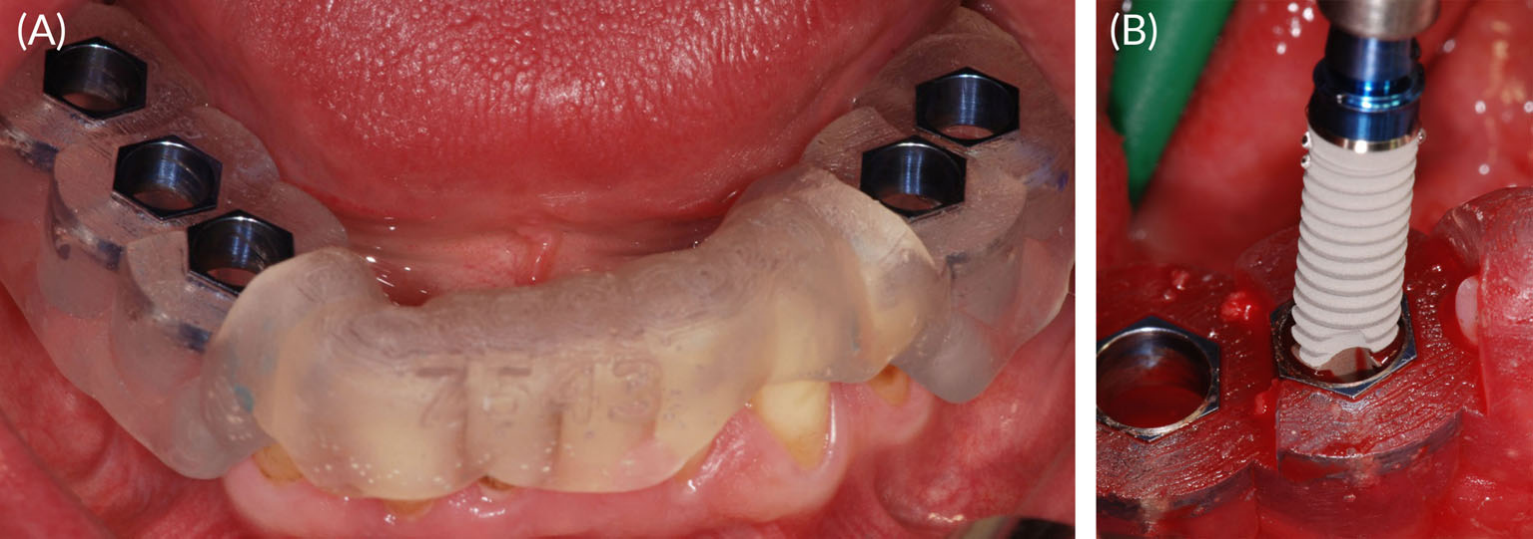
A, Surgical guide, fabricated by stereolithography and supported by the natural residual dentition. B, Guided implant placement (XiVe, Dentsply Sirona, New York). The guide facilitates optimal positioning and angulation

**Figure 2:**
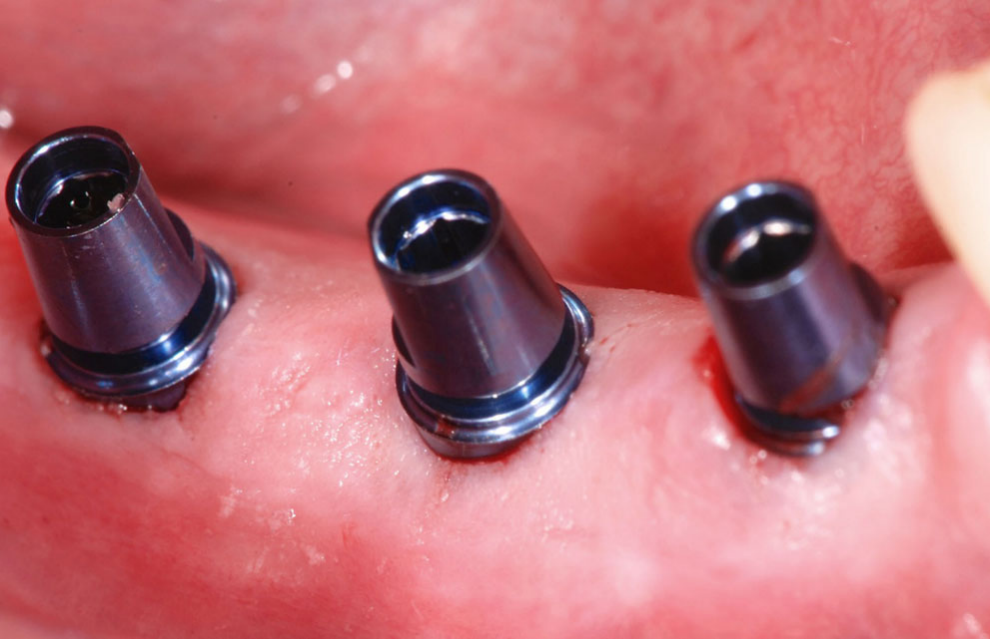
Freshly inserted implants after minimally invasive (flapless) surgery and with the temporary abutments already connected (TempBase, Dentsply Sirona, New York)

Stereolithographic tooth‐supported guides were made to transfer the 3D‐planned implant positions to the surgical situation (Figure 1A,B). A flapless punch technique was employed in 2 patients, whereas 18 patients were approached by flap surgery via a crestal incision. The same implant system was used in all patients (XiVE; Dentsply Sirona, New York) and insertion performed as per the manufacturer's drilling protocol (Figure 2). Primary stability was captured via insertion torque and Periotest values with healing abutments in place.

### 2.6 Immediate provisionalization

**Figure 3:**
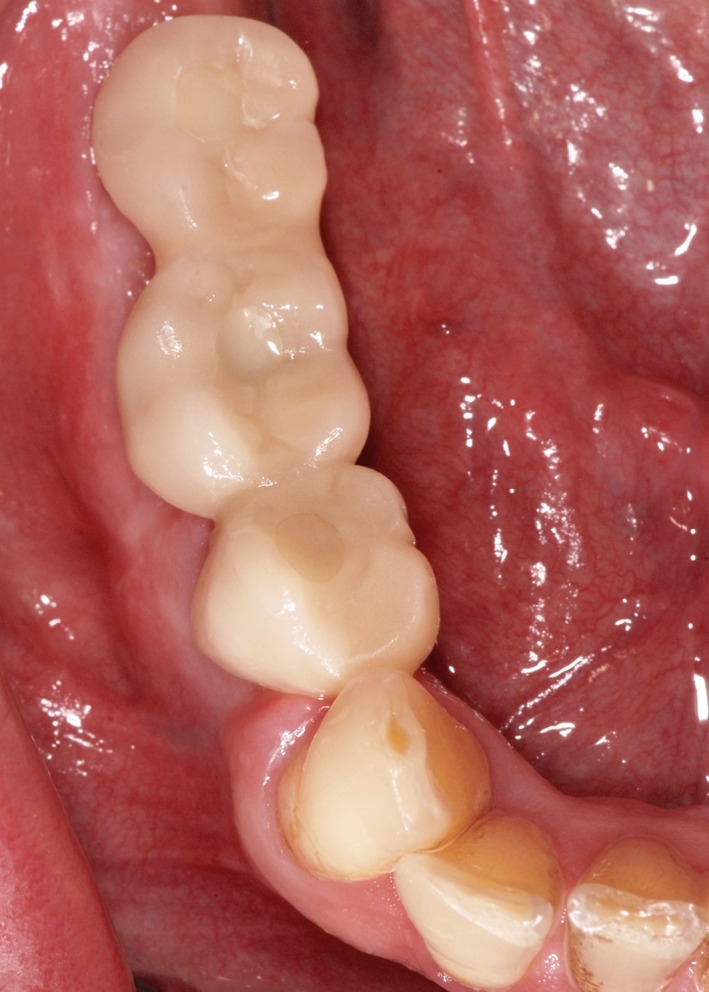
Immediate temporary restoration, consisting of three splinted and screw‐retained units of acrylic resin, 1 month after implant surgery

Single or splinted crowns were designed from resin (SR Ivocron; Ivoclar Vivadent, Schaan, Liechtenstein) for immediate delivery by screw retention on customized temporary abutments (TempBase; Dentsply Sirona, New York; ComboLign, Bredent, Senden, Germany). Screws were tightened using a ratchet as described in the user's manual until an insertion torque of 14 Ncm. They were delivered and adjusted to the randomized occlusal protocol not later than 72 hours after surgery. Any subsequent manipulations other than for occlusal adjustment, retightening loosened screws, or repair were avoided. Figure 3 illustrates an example.

### 2.7 Follow‐up examinations

**Figure 4:**
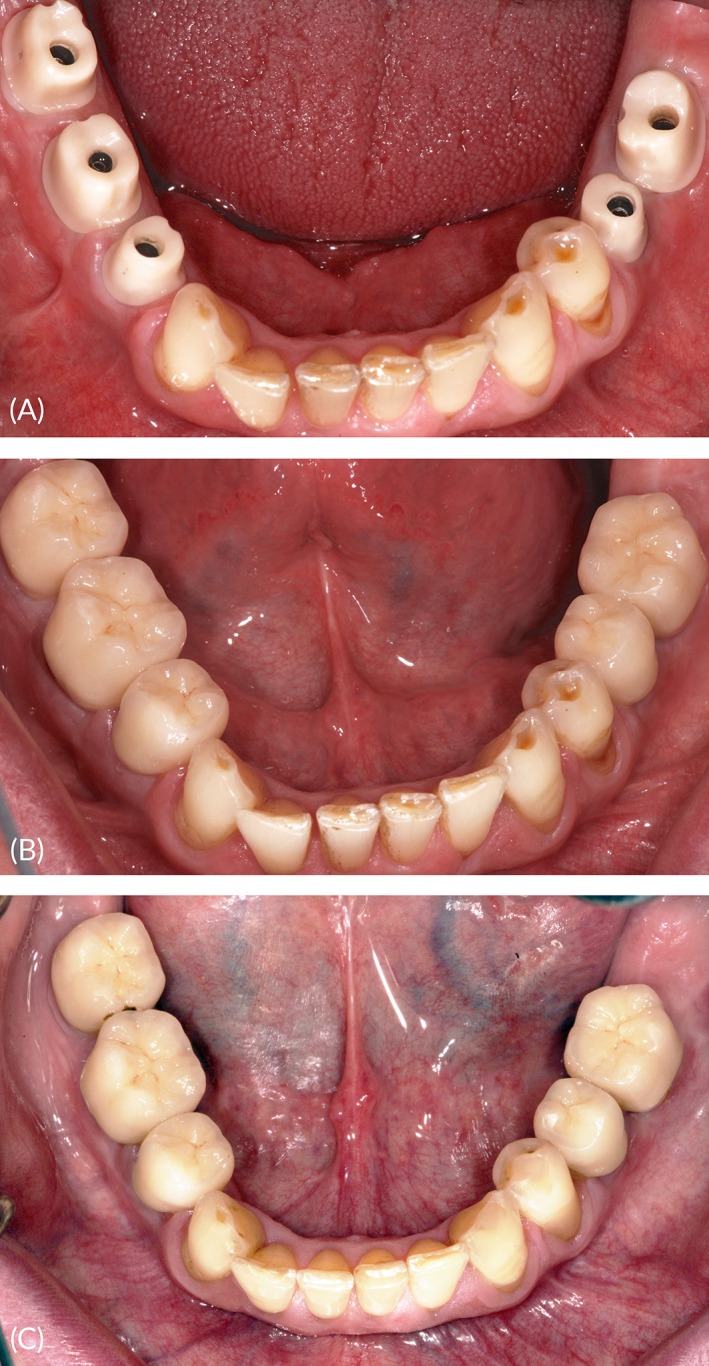
Definitive single‐tooth restorations, cemented to customized zirconia abutments, 8 and 36 months after implant surgery

One week after surgery, we recorded gingival^24^ and plaque^25^ scores and checked for signs of inflammation, necrosis, dehiscence, or pyogenic infection. Follow‐up visits were scheduled for every 4 weeks to examine the soft tissue and periapical radiographs, verify the stability of the temporary restorations, and evaluate any dental problems. The same visits were used for occlusal adjustments, including those required by the randomization protocol. At the last follow‐up, we evaluated gingival and plaque scores, Periotest values evaluated with the healing abutments after removal of the superstructures, occlusal parameters, as well as radiographic parameters. The definitive splinted or single‐tooth restorations were cemented or screwed to the implants 6 to 8 months after surgery (Figure 4A‐C). Success criteria defined by Misch et al^26^ were evaluated 12 and 36 months after implant insertion.

### 2.8 Assessment of MBDs

**Figure 5:**
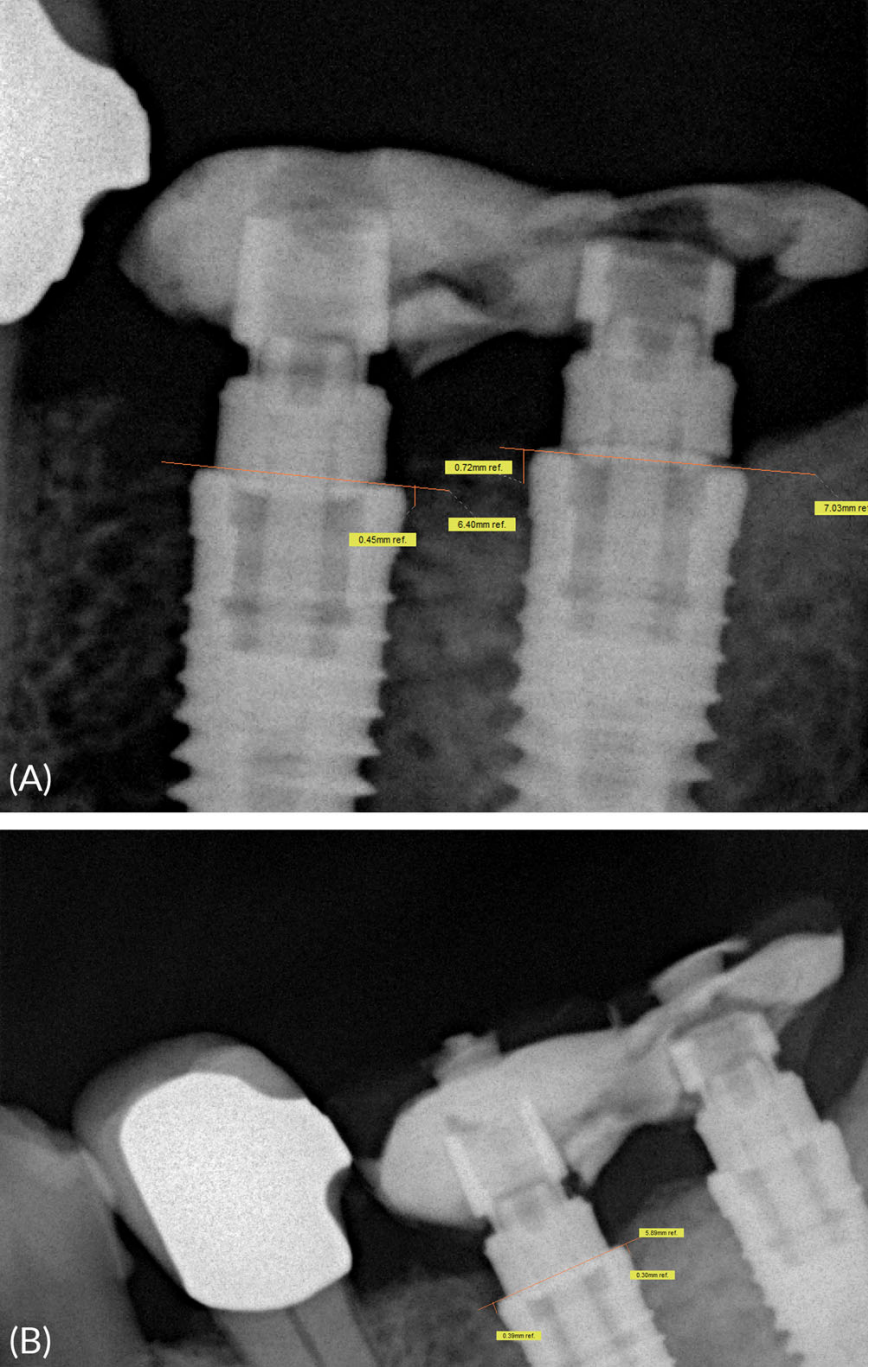
Marginal bone defects as measured in the present study, based on radiographs and using a horizontal reference line at baseline and 36 months post op

An examiner (M. S.)––not involved in the surgical and restorative procedures––assessed the MBDs based on digital perpendicular long‐cone radiographs (Sidexis, Orthophos plus DS; Sirona) obtained immediately after implant surgery (baseline) and at 1, 2, 3, 6, 12, and 36 months. Dimensions were calibrated by the known parameters of implant diameter and length. Starting from the implant shoulder, distances were measured to the mesial and distal points of distal implant‐bone contact (magnification: ×2). Following this principle, the bone loss was calculated by resetting each distance between a crestal bone level and the implant shoulder to zero from one follow‐up visit to the next (Figure 5A‐D).

### 2.9 Patient satisfaction questionnaire

Given the importance of patient‐centered outcome measures, we took advantage of the 36‐month follow‐up to evaluate patient satisfaction. Using a questionnaire that included visual analog scales, we asked each patient to rate a number of parameters that concerned any restoration he or she had worn before implant treatment, the implant surgery, the provisional restorations, and the definitive prosthetic rehabilitation. Each topic was rated for esthetics, access for oral hygiene, phonetics, and chewing comfort by visual analog scales (1 = maximum satisfaction; 10 = maximum dissatisfaction).

### 2.10 Statistical analysis

Data were analyzed using SPSS Statistics (v. 18.0; SPSS Inc., Chicago, Illinois) and Microsoft Excel (v. 2003; Microsoft Corporation, Redmond, Washington). Descriptive statistics were contributed by a biostatistician. The bone‐level data were confirmed to be normally distributed by Kolmogorov‐Smirnov testing. A general linear model with repeated measurements was used to assess the changes in bone levels between visits in both the test and the control group. Using a Friedman test, the Periotest values were analyzed at baseline and 12 and 36 months after surgery. Intergroup comparisons of patient satisfaction were performed using a Mann‐Whitney *U*‐test.

## 3 RESULTS

### 3.1 Pertinent patient data

**Figure 6:**
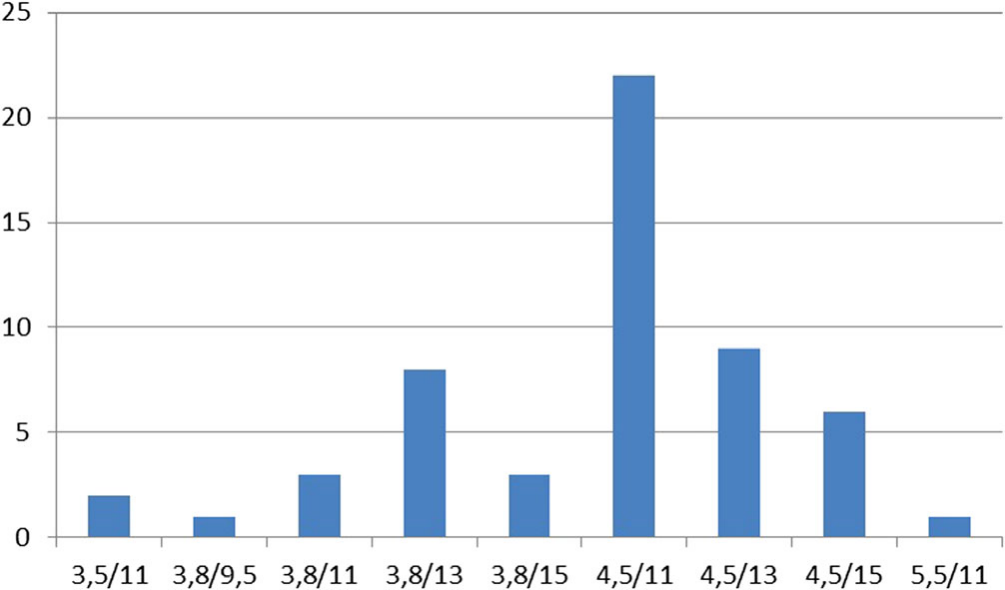
Distribution of implants inserted

We enrolled 20 patients (13 women, 7 men) aged 54 ± 11.9 years (range: 33‐70 years). One patient withdrew for personal reasons after randomization but was replaced to bring the sample back to 20. The study was open to maxillary cases, but all consecutive implants and temporary restorations were inserted in the mandible. Upper‐jaw antagonists at baseline included natural teeth in 65% (n = 13), implant‐borne restorations in 10% (n = 2), mucosa‐supported dentures in 20% (n = 4), and periodontally supported dentures in 5% (n = 1) of cases. One patient being unavailable for the last follow‐up, the entire 36‐month observation period could be analyzed for 19 patients (occlusal study group: n = 8; nonocclusal control group: n = 11) comprising a total of 52 evaluable implants. Distribution of diameter and length of the inserted implants is presented in Figure 6.

### 3.2 Implant survival and success

One implant could not be inserted due to bone deficiency, so that 58 of the 59 initially planned implants were actually placed. Three were left unrestored due to inadequate primary stability. One implant was lost 24 months after surgery and was not reinserted for prosthetic reasons; however, this implant was recorded as failure.^26^ Hence the survival and success rates were 97.1% in the control and 100% in the study group, or 98.2% based on both groups. Two implants were associated with minor bone deficiencies and three with mucositis.

### 3.3 Bone density and implant stability

**Figure 7:**
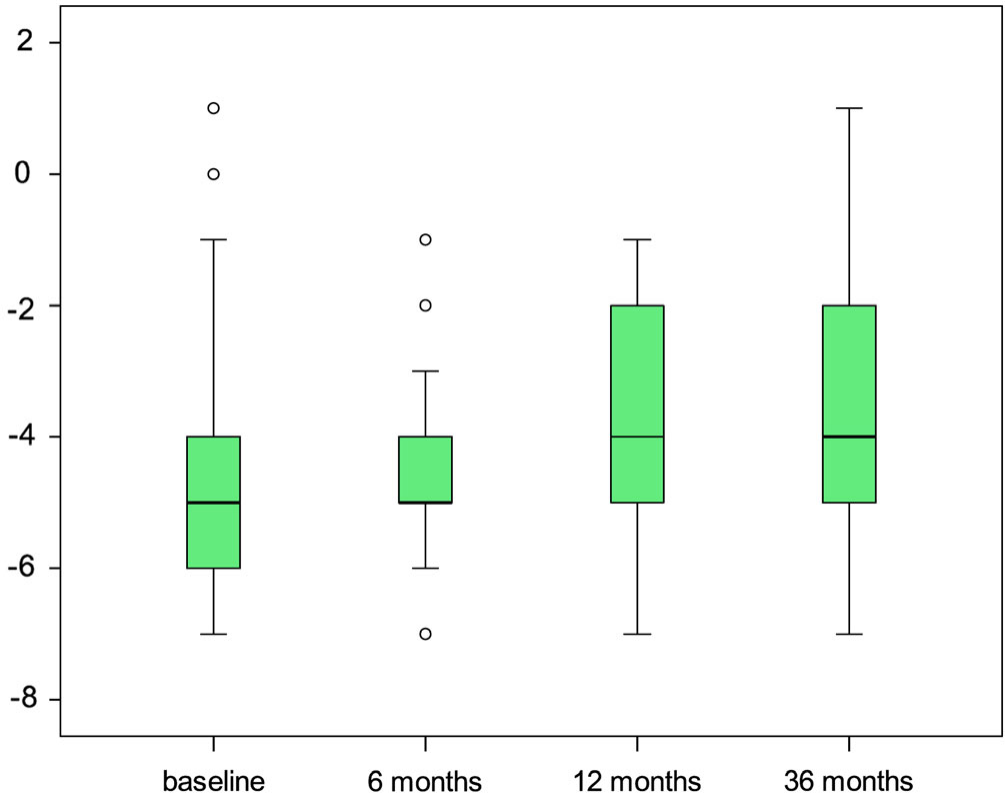
Boxplot analysis of Periotest values at baseline as well as 6, 12, and 36 months after implant surgery

Bone quality as defined by Lekholm and Zarb^27^ was D2 in 87.3% and D1 in 12.7% of cases. Mucosal biotypes were normal at 31, thin at 4, and thick at 20 implant sites. Insertion torque was >35 Ncm in 85.5%, >45 Ncm in 5.5%, and <35 Ncm in only 9.1% (n = 5) of the implants. Periotest values averaged −4.48 ± 1.66 (range: −7 to +1) at baseline, −3.98 ± 1.75 (range: −7 to −1) after 12 months, and − 3.50 ± 2.13 (range: −7 to +1) after 36 months. Figure 7 illustrates Periotest values at baseline as well as at 12 and 36 months after surgery, yielding no significant difference between the two measurements (*P* = 0.054; Friedmann test).

### 3.4 Maintenance, complications, compliance

At the 36‐month follow‐up, 50%, 35.3%, or 13.7% of patients showed plaque scores of 1, 2, or 3, respectively. Gingival index scores were 0 in 51% (n = 26), 1 in 35.7% (n = 18), and 2 in 13.7% (n = 7) of implants. All clinical evaluations were performed with healing abutments. Complications among the temporary restorations were imprecise fit (n = 3), requirements for occlusal adjustments (n = 2), fracture (n = 2), and screw loosening (n = 3). All patients in the study group and 90.1% in the control group returned for the follow up‐visits as scheduled. Restorative complications of the permanent superstructures were restricted to 1 patient with a single tooth restoration (chipping).

### 3.5 Marginal bone defects

**Figure 8:**
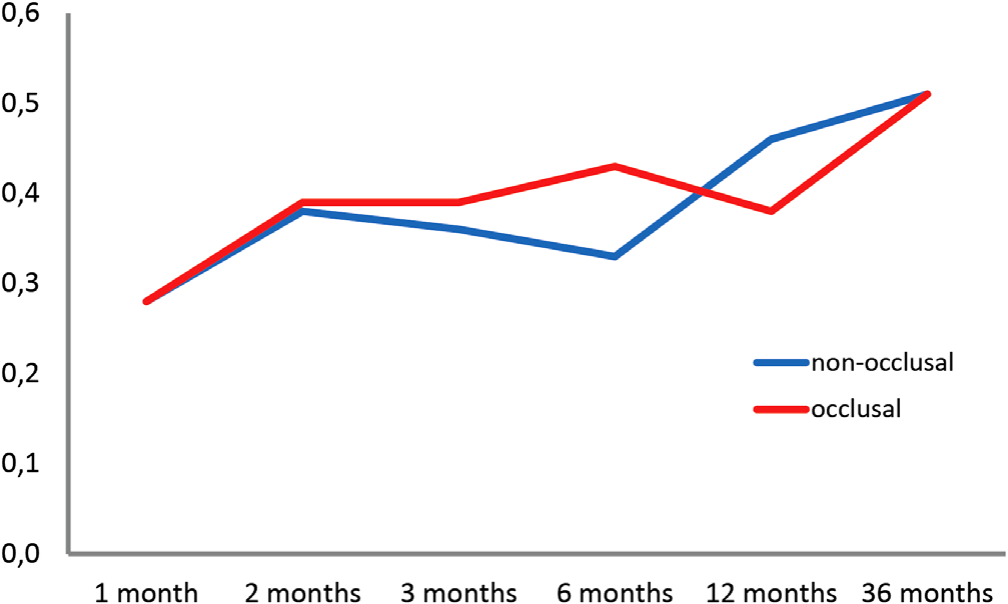
Marginal bone defects in relation to the time of implant surgery, evaluated as a general linear model with repeated measurements

**Figure 9:**
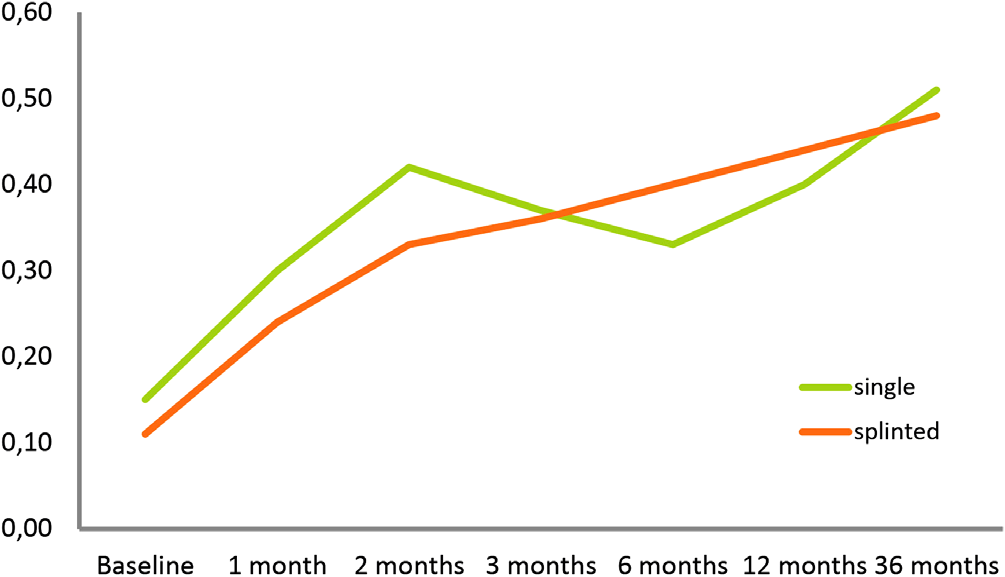
Marginal bone defects as a function of superstructure types (single vs splinted) in relation to the time of implant surgery, evaluated as a general linear model with repeated measurements

MBD data measured on radiographs were normally distributed (Kolmogorov‐Smirnov test). At baseline, these MBD measurements averaged 0.08 ± 0.16 mm (range: 0.00‐0.60 mm) in the test vs 0.16 ± 0.29 mm (range: 0.00‐1.42 mm) in the control group. At the 12‐month follow‐up, they averaged 0.38 ± 0.33 mm (range: 0.00‐1.22 mm) vs 0.46 ± 0.49 mm (range: 0.00‐2.28 mm) and, at the 36‐month follow‐up, 0.51 ± 0.43 mm (range: 0‐1.20 mm) in the test vs 0.51 ± 0.42 mm (range: 0‐1.19 mm) in the control group. Figure 8 illustrates how MBDs developed over time. Highly significant (*P* < 0.001) increases were noted in both the test and the control group but did not involve a significant intergroup difference (*P* = 0.319). A tendency for somewhat lower values in the test group was not significant (*P* = 0.803). Figure 9 illustrates these developments around single‐tooth vs splinted restorations, demonstrating that the type of superstructure did not make a difference to marginal bone levels over time (*P* = 0.180).

### 3.6 Patient satisfaction

**Figure 10:**
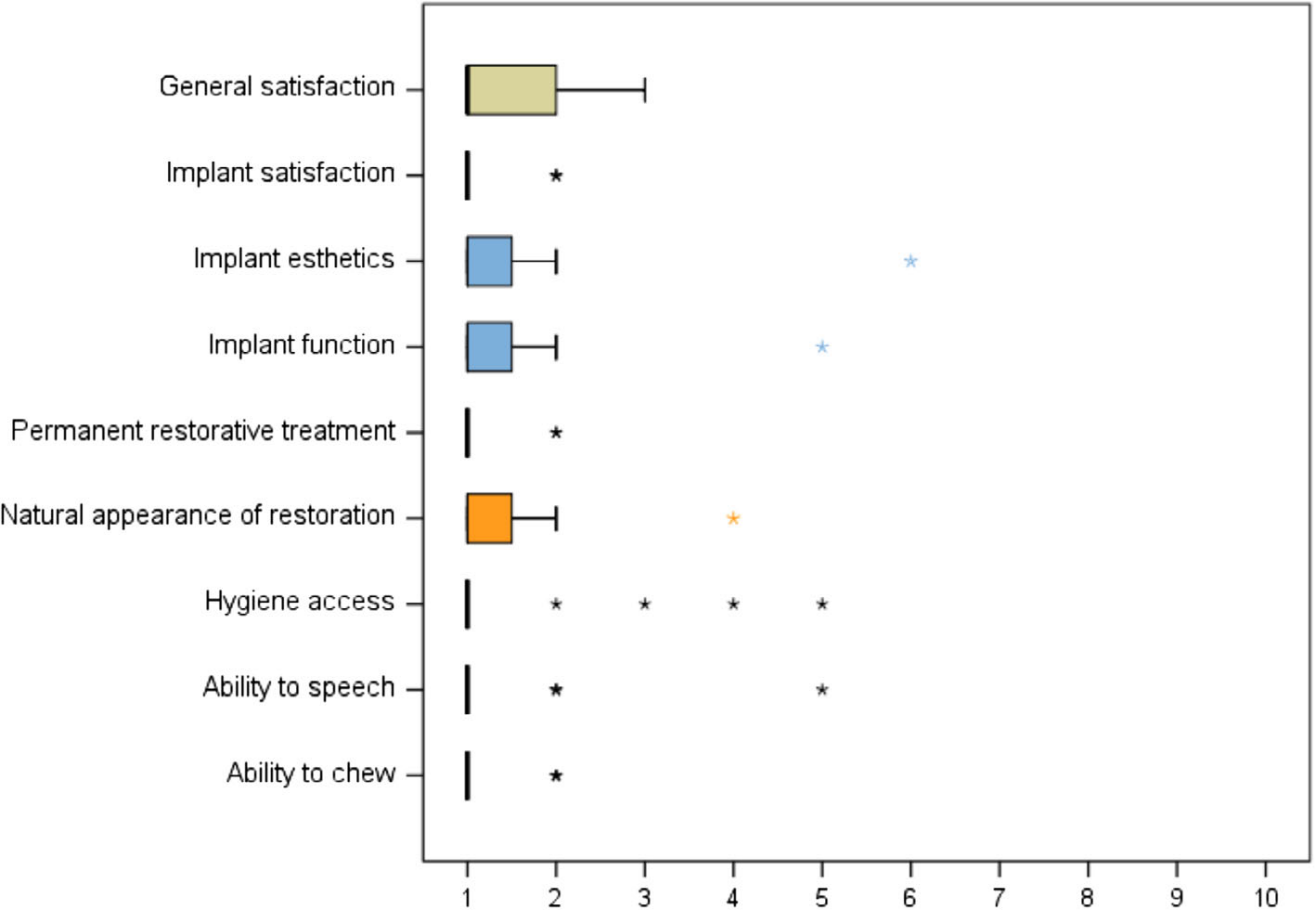
A box plot of the descriptive analysis of patient satisfaction

Fifty‐two evaluable implants were inserted to replace natural dentitions in 13 patients and fixed prosthetic solutions in 6 patients. No cases of temporomandibular disorder were noted after implant and restorative treatment. All patients (both groups) affirmed their willingness to undergo implant surgery with immediate provisionalization once again if faced with a similar situation. Patients' ratings were performed on visual analog scales (1 = maximum satisfaction; 10 = maximum dissatisfaction) and averaged 1.8 ± 2.1 (range: 1‐9) for esthetics and 1.4 ± 2.2 (range: 1‐5) for function of the immediate provisionalization. Esthetics of the definitive restorations was rated as 1.4 ± 1.6 (range: 1‐4), access for oral hygiene as 1.5 ± 2.1 (range: 1‐5), phonetics as 1.4 ± 2.2 (range: 1‐5), and chewing ability as 1.2 ± 0.5 (range: 1‐2). A box plot of the descriptive analysis is illustrated in Figure 10. None of these parameters revealed any significant intergroup differences (esthetics: *P* = 0.442; hygiene: *P* = 0.395; phonetics: *P* = 0.395; and chewing: *P* = 0.177; Mann‐Whitney *U*‐test). All patients affirmed that the implant treatment had improved their quality of life.

## 4 DISCUSSION

Recent studies have investigated potential effects of occlusion on immediate temporary restorations. It has been stated repeatedly that controlling occlusal forces is essential to successful immediate loading.^19^, ^28^, ^29^, ^30^ An extensive systematic review and meta‐analysis did not arrive at a conclusive statement about occlusal contacts during osseointegration of dental implants.^31^ Another review, indicating disagreement about occlusal guidelines in immediate loading, nevertheless recommended a concept of centric contacts only.^30^ No significant differences in survival rates, bone loss, or soft‐tissue parameters were identified in a randomized clinical study of 100 implants immediately loaded either in full occlusion or in mild infraocclusion.^4^

The sample size of our study is too small for a statistical comparison of restorations with different numbers of units. Yet our finding of no difference in survival/success rates is in contrast to a previous study of 307 implants supporting two‐ to four‐unit fixed partial dentures in 117 patients.^21^ Investigating any effects of different loading protocols on implant survival/success, implant stability quotient, insertion torque, and marginal bone levels over 2 years, implant survival was 100% with both immediate nonfunctional and delayed loading vs 93% with immediate functional loading. Seven implants, most of them in two‐unit restorations, accounted for this difference. As primary stability at insertion did not differ between the three groups, the authors of that study suggested loading‐related factors as accounting for the significantly higher failure rates in their group of immediate functional loading, and they also concluded that the number of units might influence implant stability during osseointegration.^21^

Five‐year results on immediate vs early functional loading in posterior mandibles yielded 100% implant survival and no significant differences in crestal bone loss (0.4 vs 0.8 mm), bleeding index (0.22 vs 0.25), or plaque index (0.17 vs 0.19) between both groups.^32^ A randomized clinical study of immediate functional vs nonfunctional loading yielded 100% survival in both groups and marginal bone losses of 1.59 mm vs 1.91 mm.^33^ A reported rate of implant survival even higher after immediate (100%) than delayed (93%) loading^7^ ran counter to 3‐year findings of poorer survival after immediate (85%) than delayed (100%) loading in the posterior mandible.^20^

Our 36‐month data evaluable for 19 patients with a total of 52 implants yielded an overall survival and success rate of 98.2% and MBDs at least as favorable as in previous studies.^4^, ^10^, ^11^, ^15^, ^20^, ^21^, ^33^, ^34^, ^35^, ^36^, ^37^ Both MBDs and the associated survival/success rates may be affected by various parameters after immediate loading in partially edentulous mandibles. Bone levels might depend on the load distribution between natural teeth and implants or on access for oral hygiene in splinted provisional restorations.^2^ Iatrogenic manipulation of the implant in the early phase of osseointegration might play a role.^18^ Even so, histological studies suggested clinically stable implants also in heavy smokers after immediate functional loading of implants.^10^, ^38^, ^39^ A recent split‐mouth study of immediate vs early occlusal loading in the posterior mandible, with up to 15 years of implant service, yielded intergroup differences in periodontal indices and crestal bone loss (mesial: 0.70 ± 1.09 mm vs 1.17 ± 1.27 mm; distal: 0.43 ± 1.02 mm vs 1.06 ± 1.33 mm) that were not statistically significant after 12.14 vs 12.40 years of loading.^40^

What might also apply to the present study is a suggestion by previous authors—who reported significant bone‐level reductions with both immediate and delayed loading over 3 years—that insertion depth may be increased in loaded implants.^20^ Our radiographic assessment showed that the baseline bone levels (0.08 vs 0.16 mm) were lower than we have reported previously (0.48 mm) for the same implant type,^2^ indicating an increased insertion depth for both groups in the present study. We also noted less radiographic resorption than previously^2^ in both groups from baseline to 6 and 12 months. Contributing factors remain speculative, but examples would include patient selection, guided surgery, modified platform surface, fit of restorations, or access for hygiene. This time we report a radiographic bone loss perhaps even lower than the 6‐ and 12‐month data presented by Degidi et al^4^ whose results of no significantly different survival/success rates or tissue reactions between both loading protocols are highly consistent with ours. We noted significant increases in marginal bone resorption within 12 months, no significant intergroup difference in the steepness of this curve, and similar defects (0.4 mm vs 0.38 mm) at the end of this period.

A recent multicenter study, designed as a randomized controlled trial, compared 10‐year results for immediate nonocclusal loading vs nonsubmerged early loading of implants, which included an analysis of peri‐implant bone and soft‐tissue levels.^41^ The investigators did not observe any significant intergroup differences in failed implants, failed superstructures, other complications, periimplant mucositis, peri‐implant bone loss, or soft‐tissue levels. Both groups were found to have gradually lost peri‐implant bone by a mean of 1.43 mm (immediate loading) or 1.42 mm (early loading) after 10 years, and the soft‐tissue levels had changed by 0.38 or 0.25 mm, respectively, during that time.

We observed restoration‐specific problems (resin fractures, loosening, inaccurate fit), periimplant mucositis, as well as imperfections of 3D planning and the surgical guide. All of these were readily manageable without compromising implant integration and radiographic appearance. Three implants were not evaluated as they were left to heal submerged due to rotational instability after insertion. To optimize force distribution, we splinted any adjacent implants as described in a similar way elsewhere.^20^, ^42^, ^43^ Esposito et al^31^ in their Cochrane Review, recommended immediate rather than early loading to optimize implant success, and the insertion torques which have been recommended for immediate loading range from 25 Ncm^4^ to 45 Ncm. In the present study, we recorded insertion torques <35 Ncm for 5 implants (study group: n = 3; control group: n = 2) without any notable effects on clinical outcomes 12 months after surgery. Splinting of the provisional restoration may well have prevented micromotion of these implants.

A systematic review has recently concluded that patients focus their expectations mainly on function, followed by esthetics.^44^ There was insufficient evidence to determine possible outcomes of immediate, immediate‐delayed, or delayed loading with regard to patient satisfaction, so that the pros and cons of different loading protocols may vary in this regard.^44^ Another review has concluded both that high patient satisfaction is the most important advantage of immediate over conventional loading and that this statement is especially true of the early healing phase.^43^ In accordance with our own findings, a recent study showed that patient satisfaction did not differ by gender, number of implants, survival, complications, and time in situ.^45^

Our finding that occlusal and nonocclusal immediate restorations did perform equally well in partially edentulous posterior mandibles is consistent with two studies^4^, ^11^ but not with others.^20^, ^21^ The 100% survival/success rate in our study group, with no major complications posing a risk to the implants, may have been due to our careful patient selection, presurgical 3D‐planning and precise intraoperative transfer, no delivery of restorations on implants with reduced primary stability, accurate laboratory and restorative procedures, as well as rigorous postoperative follow‐up. We also noted no differences between the single‐unit and the splinted multi‐unit restorations, and plaque and mucosal parameters were well within the range of similar reports.^3^, ^46^

Patients benefit from immediate loading in several ways. They are not only subjected to less chair time, no second‐stage surgery, and a shorter healing period, but they also save money. Another point in favor of patient acceptance is the postoperative comfort of not having to wear a removable denture.^2^, ^47^, ^48^ Given that even a “high‐load” scenario like the posterior mandible does not seem to affect the osseointegration of screw‐type implants, a case could even be made for immediate definitive restorations. That being said, temporary restorations made of resin still have their advantages in the initial phase of healing and osseointegration: they minimize the requirements for laboratory fabrication and soft‐tissue conditioning and, even more importantly, facilitate the use of definitive single‐tooth restorations in the mandible.

## 5 CONCLUSIONS

No clinically relevant differences in MBDs were observed between loaded and nonloaded immediate restorations in partially edentulous posterior mandibles up to 36 months. Within the limitations of our study, both treatment options can be considered a viable treatment concept in selected patients. Randomized controlled trials are needed to disclose any kind of superiority of either protocol in specific situations or jaw areas.

## AUTHOR CONTRIBUTIONS

All authors made (a) substantial contributions to the conception and/or design of the work; or the acquisition, analysis, or interpretation of data for the work; and (b) drafted the article or revised it critically; and (c) approved the final version of this manuscript; and (d) agreed to be accountable for all aspects of the work.
